# The Appearance of Plague

**Published:** 1919-05-31

**Authors:** 


### THE APPEARANCE OF PLAGUE.

Elsewhere in this issue we publish the official announcement of the reappearance of a bubonic plague in this country, and it may be advisable that the public and every one interested in the health of communities should recall the present state of our knowledge in regard to the spread of this disease.

Plague is now considered to be essentially a disease of rats, which occasionally attacks man, fleas and bugs providing the means of active transference. If opportunity does not occur for fleas from infected rats to gain access to human beings, it is stated that the disease will then never extend. The elementary principles of cleanliness form a very obvious line of preventive measures, but at the same time one knows the regrettably dirty conditions of slums in this country, which have long prevailed in spite of innumerable attempts at organised improvement. The situation may therefore well suggest an alarming prognosis.

There are, however, several points in this connection which may somewhat modify our opinion. Observations in India show the relative rarity of direct infection. The plague bacillus has probably a very short extra-corporeal existence, and hence dust is never a serious or lasting source of infection. Even allowing the frequency of parasitic bites in this country, it must be remembered that during the great epidemic plague of the seventeenth century it was the black rat which was chiefly prevalent, whereas nowadays invasion by the Norwegian or brown rat has almost completely replaced the smaller black species. The common brown rat is a relatively shy animal as far as the average homes of this country are concerned, but in India, where the disease is endemic, the black rat lives and breeds in the houses in close, undisturbed proximity to human beings.

Rat extermination in England, expensive though it has been, has reached a stage of efficiency which allows, with relatively little extra restriction and energy, prevention of any extensive transference by means of fleas coming directly from an infected animal. Human contacts are, it is stated, not a serious consideration in England, for they are readily controlled under simple sanitary administration. The present situation need not as yet be publicly considered another alarming disease invasion of this country.

